# Funders’ responsibility to ensure value in research: a self-audit by the Health Research Board Ireland

**DOI:** 10.12688/hrbopenres.13224.1

**Published:** 2021-04-09

**Authors:** Anne Cody, Maura Hiney, Patricia Clarke, Mairead O'Driscoll

**Affiliations:** 1 Health Research Board, Dublin, DO2 H638, Ireland

**Keywords:** Research funding, funding policy, research standards, research design, self-assessment

## Abstract

As a public funder of health research, the Health Research Board (HRB) Ireland has an obligation to manage its funds well and to maximise the value of the research that it funds. Ways in which research funding can be wasted have been examined by researchers over the years, and a seminal series on research waste was published in the Lancet in 2014. The series systematically analysed every step of the funding lifecycle in five major stages and made recommendations to various actors including research funders.

Prompted by its participation in the Ensuring Value in Research Funders’ Forum, between June and October 2019 the HRB undertook a self-audit against the 17 recommendations and 35 sub-recommendations identified in the Lancet series. Key HRB staff collated relevant policies and practices regarding each recommendation and sub-recommendation and assessed the HRB’s performance under each heading. The self-assessment reflects the state of HRB policies and practices in October 2019. Of the 17 recommendations, two were found not to apply to the HRB. Of the remaining 15 recommendations covering 33 sub-recommendations, five were found to be areas of strength and six were found to be areas of partial strength. These 11 recommendations encompass 22 sub-recommendations. Areas of strength reflect work over many years such as support for evidence synthesis, strong processes around award selection, driving research integrity and open data including an HRB-funded open publishing platform.

Four recommendations were found to be areas for growth. These mostly revolve around real time reporting of study protocols and of ongoing funded research outside of clinical trials. Work is progressing to address some of these areas.

## Introduction

Funders of health research typically receive money from members of the public directly in the case of charitable organisations or via taxation in the case of state agencies. Funders have an obligation to make best use of this income, to maximise its societal benefits. Research waste occurs when the use of money and/or effort is not optimal. Ways in which research waste can occur have been examined by researchers over the years ( Chalmers & Glasziou, 2009), leading to the publication in 2014 of a series in the Lancet, put together by Chalmers and Glasziou, ( Chalmers *et al.*, 2014), ( Ioannidis *et al.*, 2014), ( Salman *et al.*, 2014), ( Chan *et al.*, 2014) and ( Glasziou *et al.*, 2014) that systematically analysed every step of the funding lifecycle in five major stages. These include 1) funding research that is relevant to knowledge users, 2) ensuring appropriate design, methods and analysis, 3) efficient research regulation and management, 4) information about the research being fully accessible, and 5) unbiased and useable research reports. It spelled out recommendations for funders, researchers, research performing institutions, publishers, policy makers, regulators and research ethics committees.

Individual funders across the world have grappled with how best to optimise the use of their funding for a long time. This is a complex and multi-faceted endeavour where best practice shifts over time. In 2017 a group of funders from different countries, including the Health Research Board (HRB), came together under the banner of the ‘ Ensuring Value in Research Funders’ Forum’ to work together on approaches to optimising the use of their funding ( Chinnery *et al.*, 2018). This is a place where health research funders of all sizes and with different remits can share ideas, learn from each other, collaborate and create impact. In 2019 the HRB undertook a self-assessment of its own practices and policies in a systematic way.

The HRB is the main funder of health research in Ireland, and a statutory agency under the Department of Health. The HRB funds research across a wide spectrum from patient-oriented and clinical research to population health sciences and health services research. Funding is provided for project and programme grants, career development, infrastructures and networks.

The HRB has an annual funding envelope of €45 million and manages 350 active awards with a total value of €240 million. Its funding Directorate includes approximately 27 staff. The HRB sees itself as a learning organisation with a tradition of leading in the development of best and next practice in specific policy areas. The HRB has three other functions: the Evidence Centre develops evidence synthesis products for the Department of Health; the National Health Information Systems provide information for health service planning and makes its data available for research; and the nascent regulatory function of the HRB encompasses the Health Research Consent Declaration Committee and a National Research Ethics Committee.

## Methods

For comparability the methods used for the HRB self-audit mirrored those reported previously by the Patient-Centered Outcomes Research Institute (PCORI, USA) ( Whitlock *et al.*, 2019) and are informed by those used by National Institutes of Health Research (NIHR, UK) for a similar exercise (M. Westmore, personal communication). Both PCORI and NIHR are members of the Ensuring Value in Research Funders’ Forum.

In total, four HRB members of staff (all authors) were chosen to participate in this self-audit based on their role in the organisation: Head of Pre-Award, Head of Post-Award and Evaluation, Programme Manager Policy and EU Funding, and Director of Research Strategy and Funding, respectively. All roles include a remit for policy. Jointly they are familiar with the spectrum of HRB funding policies, most of which have been driven personally by these individuals, and the national and international discussions informing these policies. The first author suggested the concept of a self-audit, the composition of the study team, methodology and respective roles at a team meeting. Approval for the project was given by the Director of Research Strategy and Funding. Work was completed as part of the overall work of the study team members for the HRB, without any additional compensation.

The group examined HRB’s existing policies, initiatives and practices against 17 recommendations for funding agencies from the *Lancet* series. Many of the recommendations contain a number of sub-recommendations to capture multiple dimensions, leading to a total of 35 areas to assess ( Table 1).

**Table 1.** Assessment of HRB’s policies and practises related to ensuring value in research.

Lancet series recommendations	Related processes or initiatives at HRB	Self-assessment agreed
Questions are relevant to users of research		
1. More investigations into research should be done to identify factors associated with successful replication of basic research and translation to application in health care, and how to achieve the most productive ratio of basic to applied research	The HRB funds basic research only in limited circumstances and as a result has a very small portfolio of basic research awards.	4. Not applicable
2. Research funders should make information available about how decisions are made about what research to support (2a) and fund investigations into the effects of initiatives to engage potential users of research in research prioritisation (2b)	a) HRB strategy contains a high degree of detail a) HRB website section called 'before you apply’ setting out the funding process a) Call guidance notes describe in detail the assessment criteria and the details of the assessment process for each call a) Members of the public provide public reviews for some schemes on the Public and Patient Involvement (PPI) aspects of applications a) Members of the public have been part of a small number of selection panels as PPI panel members a) Some external observers attend panel meetings and gain first-hand experience with the process for sharing with the wider community b) HRB schemes are typically open to many types of research. Schemes serving the research needs of the Irish health and social care system require a knowledge user lead applicant and a researcher lead applicant	2. Area of partial strength – practices reasonably or partially address all sub- recommendations
3. Research funders and regulators should demand that proposals for additional primary research are justified by systematic reviews (3a), showing what is already known (3b) and increase funding for the synthesis of existing evidence (3c)	a) Requirement of systematically gathered evidence ((1) systematic identification of previous work, 2) critical appraisal, 3) synthesis of the evidence and 4) interpretation of findings) in growing number of schemes b) Applications for clinical trials requests search of relevant registries c) HRB has covered a national subscription to the Cochrane Library since 2002 (as first jurisdiction in the world jointly with Northern Ireland) c) HRB has funded systematic review training for approx. 90 2-year Cochrane Fellowships and short courses for approx. 1,000 participants 2002–2018 c) Consolidated training for evidence synthesis across various methodologies in Evidence Synthesis Ireland since 2018, also acts as Cochrane Ireland c) HRB funds evidence synthesis service for the National Clinical Guidelines Group c) HRB Evidence Centre provides evidence synthesis products to Department of Health to inform policy making	1. Area of strength – practices reasonably address all sub-recommendations
4. Research funders and research regulators should strengthen and develop sources of information about in progress research (4a), ensure that this information is used by researchers (4b), insist on publication of protocols at study inception (4c), and encourage collaboration to reduce waste (4d)	a) Awards published on website and open government data portal. HRB Open Research encourages authors to register their systematic reviews. b) Only clinical trials required to register protocols; HRB provides infrastructure to publish protocols for any study (HRB Open Research). c) HRB is funding some network awards and participating in some relevant policy initiatives at national and EU level. d) HRB active in a number of relevant policy initiatives	3. Area of growth - practices do not address all sub- recommendations, either reasonably or partially
**Appropriate research design, conduct, and analysis are employed**		
5. Make publicly available the full protocols (5a) analysis plans or sequence of analytical choices (5b) and raw data (5c) for all designed and undertaken biomedical research	a) HRB led development of National Research Integrity Policy, which stipulates data sharing and open publication as good research practices b) Providing infrastructure for publishing protocols/analysis plans (HRB Open Research) c) FAIR (Findable, Accessible, Interoperable and Reusable) data steward pilot c) Open access policy c) HRB Open Research endorses FAIR Data Principles, alongside an Open Data policy, as a framework to promote the broadest reuse of research data. c) HRB Data Management and Sharing policy and data management plan template c) All articles in HRB Open Research include the source data underlying published results	3. Area of growth - practices do not address all sub- recommendations, either reasonably or partially
6. Maximise the effect to bias ratio in research through: defensible design and conduct standards (6a), a well-trained methodological research workforce (6b), continuing professional development (6c), and involvement of non-conflicted stakeholders (6d)	a) High quality panel composition and call guidance b) HRB led development of National Research Integrity Policy, addresses training in good research practices, including methodology, design and Good Laboratory Practice (where appropriate), at all career stages b and c) Significant funding for methodological support, infrastructures and research c) FAIR data steward training pilot c) HRB supporting national pilot of on-line training in research integrity and good research practice d) Panels members and reviewers are not based in Ireland d) PPI initiatives at funding decision making d) Unconscious bias briefing for each selection panel d) Conflict of Interest rules	1. Area of strength – practices reasonably address all sub-recommendations
7. Reward (with funding and academic or other recognition) reproducibility practices and reproducible research and enable an efficient culture for replication research	HRB does not support the replication of research through funding. However HRB enables a culture supporting the replication of research via publishing in HRB Open Research and encouraging research outcomes that are re-useable through data standards.	3. Area of growth - practices do not address all sub- recommendations, either reasonably or partially
Research regulation and management is efficient		
8. People regulating research should use their influence to reduce other causes of waste and inefficiency in research	HRB does not regulate research	4. Not applicable
9. Regulators and policy makers should work with researchers, patients, and health professionals to streamline and harmonise the laws, regulations, guidelines and processes that govern whether and how research can be done (9a), and ensure that these factors are proportionate to the plausible risks associated with the research (9b)	a and b) HRB worked with Department of Health to make new Health Research Regulations (HRR) proportionate. HRB hosts the HRR Consent Declaration Committee and is taking on the National Ethics Committee function. a and b) HRB is a member of Science Europe legislation working group, signatory of the Declaration On Research Assessment (DORA), and has supported various lobbying efforts a and b) HRB contributes to strategic planning of Horizon Europe regulations, and the preparation of Horizon 2020 work programmes a and b) HRB is a member of national fora (National Open Research Forum and National Research Integrity Forum) that are working to develop national policies	1. Area of strength – practices reasonably address all sub-recommendations
10. Researchers and research managers should increase the efficiency of recruitment and retention of participants, data monitoring, and data sharing in research through the use of research designs known to reduce inefficiencies, (10a), and do additional research to learn how efficiency can be increased (10b)	a) and b) HRB funds Trials Methodology Research Network to carry out research and training in recruitment and retention, good practice in clinical trials etc. Also funding Studies Within A Trial for intervention studies. a) HRB Clinical research governance framework addresses data monitoring a) HRB is a member of the Irish National ORCID (Open Researcher and Contributor ID) Consortium a) HRB funding proof of concept for secure sharing and linkage of research data in line with best international practices	2. Area of partial strength – practices reasonably or partially address all sub- recommendations
11. Everyone, particularly individuals responsible for health-care systems, can help to improve the efficiency of clinical research be promoting integration of research in everyday clinical practice	HRB information systems (Drugs and Alcohol, mental health) used to drive service decisions HRB Evidence Centre provide evidence synthesis products to Department of Health to inform policy decisions HRB funding and dissemination activities Key individuals in health service are involved in Horizon 2020/ FP7 projects, and in EU/ international committees. HRB involved in Department of Health Group preparing Health Information Strategy Health Research Forum to be set up by DOH with national healthcare provider, HRB and other stakeholders	2. Area of partial strength – practices reasonably or partially address all sub- recommendations
All research is reported and data are accessible		
12. Institutions and funders should adopt performance metrics that recognise full dissemination of research (12 a) and reuse of original datasets by external researchers (12b)	a) HRB application forms ask for dissemination of previous research across multiple formats (including policy/practice influence and general public) a) HRB applies the Payback framework for collecting a broad range of metrics a) HRB Open Research provides support for full dissemination of research b) HRB National Health Information Systems are accessible to researchers b) Dedicated funding call for Secondary Data Analysis Projects b) Various initiatives to support good management of data created in HRB-funded research to facilitate re-use (member of Science Europe Group that is driving voluntary alignment of Research Data Management planning and its assessment across Europe, Training of FAIR data stewards, FAIR data training)	2. Area of partial strength – practices reasonably or partially address all sub- recommendations
13. Investigators, sponsors, regulators, research ethics committees, and journals should systematically develop and adopt standards for the content of study protocols (13a) and full study reports (13b), and for data sharing practices (13c)	a) and b) HRB Open Research supports a Registered Reports tool (review of published Study Protocols before data is collected followed by full published study as a Research Article) b) HRB Open Research adheres to various international standards c) HRB Policy on Research Data Management and Sharing in place for all grants issued from 1 January 2020 c) Member of Science Europe Working Group driving voluntary alignment of Research Data Management and its assessment across Europe c) HRB subscribes to the FAIR principles (supporting FAIR data steward training and conducting a FAIR data pilot)	1. Area of strength – practices reasonably address all sub-recommendations
14. Investigators, sponsors, regulators, research ethics committees, journals and legislators should endorse and enforce study registration policies (14a), wide availability of full study information (14b), and sharing of participant-level data for all health research (14c)	a) HRB requires grantees of regulated and non-regulated clinical trials to register awards in publicly accessible register a) and b) HRB Open Research promotes Registered Reports, registration in in appropriate register (e.g. clinicaltrials.gov, PROSPERO) b) HRB provides grant data to the Government Data portal. HRB posts data on grants on HRB website but not with metadata.	3. Area of growth - practices do not address all sub- recommendations, either reasonably or partially
Research reports are complete, unbiased and useable		
15. Funders and research institutions must shift research regulations and rewards to align with better and more complete reporting	HRB Clinical Research governance framework requires full reporting HRB has signed up to DORA and is using a wide range of potential output formats for assessment of applicants’ track record HRB has been instrumental in shaping the National Open Research Framework which promotes: - information on open research and associated skill attainment in national level research reporting and evaluation - support and reward for researchers within the academic career system who participate in a culture of sharing their research results - adoption of 'responsible metrics' by Funders and institutions and rewarding the full diversity of outputs and of recording the broader social impact of research ('next-generation metrics') HRB leading various national discussions on cultural shift to better regulations and rewards e.g. GDPR, Ethics, research integrity, promotion of DORA, conference presentations etc.	2. Area of partial strength – practices reasonably or partially address all sub- recommendations
16. Research funders should take responsibility for reporting infrastructure that supports good reporting and archiving	HRB has own publishing platform for various types of articles and datasets (HRB Open Research) HRB has a suite of policies on Open Access, data management etc and has worked nationally and internationally to determine best way of making data FAIR	1. Area of strength – practices reasonably address all sub-recommendations
17. Funders, institutions and publishers should improve for authors and reviewers the capacity for high-quality and complete reporting	HRB supports general FAIR data training, the development of expert data stewards and a pilot to make data on funded projects FAIR HRB Open Research provides: - support for authors for various reporting options; -provision of Registered Reports tool; -citation for peer-reviewers; -advice on how and where to link publications and underlying data. The National Open Research Framework provides: -clarity on open access publication issues -recognition of the need for standardised and accredited skills for open research at all career levels HRB is working with international partners to deliver online DMP template.	2. Area of partial strength – practices reasonably or partially address all sub- recommendations

To ensure that diverse perspectives were captured, initially each author collated relevant HRB materials, policies, or practices of which they were aware in their area of expertise. This material was collated by the first author, shared with the other authors and then added to collectively. The scope of the material to be included under each recommendation was agreed jointly.

On this basis, each author then independently categorised fidelity to the 17 recommendations as: 1) “area of strength” – HRB’s practices reasonably address all sub-recommendations; 2) “area of partial strength” – HRB’s practices reasonably or partially address all sub-recommendations; 3) “area of growth” – HRB’s practices do not address all sub-recommendations, either reasonably or partially; or 4) not applicable. These rating were used by PCORI in their self-audit. Discrepancies were resolved through discussion and final ratings reflect consensus.

The kick-off meeting took place in the HRB offices in June 2019, with in an depth discussion of the methodology and approach. Two more meetings took place in July to finalise the collation of materials feeding into the self-audit. The individual assessments of HRB performance against the recommendations were completed in October 2019 with good overlap between authors. Any differences in rating were resolved at a meeting in October 2019. The wording for publication of the HRB related processes or initiatives was finalised and agreed in February 2020.

## Results

The self-assessment reflects the state of HRB policies and practices in October 2019. It adopted a whole-of-organisation approach beyond the HRB funding remit.

Table 1 sets out the results across the five key stages of research: (1) relevance of questions; (2) appropriate design, conduct and analysis; (3) efficient regulation and management; (4) full reporting and accessible data; and (5) complete, unbiased and useable reports. Of the 17 recommendations, two were found not to apply to the HRB (1 and 8) due to its remit. Of the remaining 15 recommendations covering 33 sub-recommendations, five were found to be areas of strength (3, 6, 9, 13 and 16) and six were found to be areas of partial strength (2, 10, 11, 12, 15 and 17). These 11 recommendations encompass 22 sub-recommendations.

Four recommendations were found to be areas for growth (4, 5, 7 and 14).

## Discussion

By nature, any self-audit has the potential for bias. We aimed to avoid bias by using an external framework that had been published previously, including contributors with different perspectives, and concluding multiple rounds of discussion and feedback on each recommendation and its sub-recommendations.

This self-assessment positively highlighted areas where significant effort has been made over many years. The areas that scored well have been a core part of the HRB work programme for some time: quality of the scientific peer review process in all its aspects, in-house and extramural support for evidence synthesis and methodological support for researchers.

For example, in early 2018 the HRB launched its own open publishing platform, HRB Open Research, that facilitates immediate publication followed by open peer review and combined with an open data policy. This plays an important role in strengthening the assessment around the reporting-related recommendations.

The role of the HRB in the implementation of Irish legislation accompanying general data protection regulation (GDPR) in the context of health research is also reflected positively. Since the self-assessment, the HRB has hosted and managed a national research ethics committee for coronavirus disease 2019 (COVID-19)-related research with expedited turnaround times for decisions, and national research ethics committees for clinical trials of regulated medicinal products and of medical devices are in imminent.

As the main funder of health research in a small country, the HRB typically issues funding calls that focus on the type of outcome expected, not on the subject area. We therefore emphasise collaboration between researchers and knowledge users in a variety of funding schemes to ensure the relevance of research findings.

The HRB has been very active in the international and national development of research integrity, FAIR data and open research, including the development of coordinated policy and frameworks. Training in research integrity is mandatory for recipients of funding and their teams, and HRB contributes to a national subscription to an online training platform for research integrity. In Ireland the HRB has played a leading role in the development of PPI capacity with innovative and new system-wide approaches.

Progress has been made in all these areas, but there are still opportunities to further improve the implementation of policies across the spectrum and enhance institutional and researcher capacity to ‘do the right thing’. The authors view the undertaking of self-audits as part of continuous improvement efforts. For example:

• The culture change required for the meaningful inclusion of members of the public across the research endeavour has only started. The HRB introduced public reviews for some funding schemes in 2017 and is providing infrastructure support for institutions to enhance their capacity for PPI, but more researchers and PPI contributors need to gain more direct experience.
• Having a route via HRB Open Research to publish protocols and outcomes quickly and without publication bias is important, but alone does not guarantee that they are published. In a recent call for COVID-19 research the publication of protocols was mandatory, which was fully implemented. We are reflecting on this experience and starting to roll out such a requirement across other schemes. There is a noticeable increase in study protocol publications on HRB Open Research across other funded projects.
• A current area of focus is around data, encompassing the review and publication of data management plans, the further broadening of FAIR data capacity through the training of data stewards in institutions, and a proof of concept initiative facilitating the safe linkage of datasets and secondary use of data. This is an evolving area internationally and capability and capacity are currently limited in Ireland.

Whilst work is ongoing in some of the four areas for growth, it is currently not clear how to address some sub-recommendations. Some areas are challenging for many funders including the HRB. The registration and real time reporting of ongoing funded research (particularly outside of clinical trials), which is captured in recommendations 4, 5 and 14, poses difficulties, with few suitable repositories. In their self-audit PCORI note similar challenges in this space ( Whitlock *et al.*, 2019). This is an area that requires more consideration in the future and would benefit from infrastructural solutions beyond the remit of the HRB.

The framework used here included recommendations for all players within the research ecosystem. It is relevant to research funding organisations but not specifically tailored towards them. Based on the experience of their respective self-audits, HRB and PCORI are currently contributing to the development of a new tailored tool for the self-assessment of research funding organisations by the Ensuring Value in Research Funders’ Forum. This will align with the Ensuring Value in Research principles, provide guidance on methodology and areas to consider, and better focus the work associated with a self-audit.

## Data availability

All data underlying the results are available as part of the article and no additional source data are required.
