# Supplementary material for: Funders’ responsibility to ensure value in research: a self-audit by the Health Research Board Ireland
Source: HRB Open Res. 2021 Jul 29;4:35. Originally published 2021 Apr 9. [Version 2] doi: 10.12688/hrbopenres.13224.2 (PMC8080938; doi:10.12688/hrbopenres.13224.2)
Supplement: Peer review history

# Peer review: Reviewer response for version 1

**DOI:** 10.21956/hrbopenres.14382.r29270

**Published:** 2021-04-27

**Authors:** Mona Nasser

**Affiliations:** 1 Plymouth Institute of Health and Care Research, Peninsula Dental School, University of Plymouth, Plymouth, UK

In order to be able to reproduce the data, the readers need to know more about, "...initially each author collated relevant HRB materials, policies, or practices of which they were aware in their area of expertise." - Either how they identified/searched them or a list of these documents (if not all of them is possible, at least some examples of them).

**In Table 1:** It would be useful that in the self- assessment that you would differentiate between transparency around having criteria and process in place on how decisions are made (which a lot of funders do) versus transparency around how those procdess are implemented e.g. minutes of meeting or external observants in the room (which less often done by funders).

**Table 1, row 3 item (a):** For this, "Requirement of systematically gathered evidence ((1) systematic identification of previous work, 2) critical appraisal, 3) synthesis of the evidence and 4) interpretation of findings) in growing number of schemes", is it possible to give a more accurate number than growing number of schemes or at least an estimate of how many schemes or applications that applies to. It will be also useful to highlight whether it is for researcher-driven grants or commission based one too or both?

**Table 1, row 3 item (b):** "Applications for clinical trials requests search of relevant registries" - Is that part of a systematic review or separate?

**Table 1, row 4 item (d):** "HRB active in a number of relevant policy initiatives" - can you give example of one initiative?

**Table 1, row 6:** can you explain what "FAIR data steward training pilot" is? You talk about FAIR data in the text but don't explain what it is.

**Table 1, row 11: **"...responsible for health-care systems, can help to improve the efficiency of clinical research be promoting integration of research in everyday clinical practice" - I assume it is improve the efficiency of clinical research "by" promoting integration?

 Consistency in using the abbreviation - In this sentence, "HRB involved in Department of Health Group preparing Health Information Strategy Health Research Forum to be set up by DOH with national healthcare provider, HRB and other stakeholders", if you use DOH as abbreviation might be useful to add after department of health (DOH).

 It wasn't clear to me how (HRB funding and dissemination activities) addressed item 11.

 You have DMP in the table 1 as abbreviation but not written as full in other parts.

Is the work clearly and accurately presented and does it cite the current literature?

Yes

If applicable, is the statistical analysis and its interpretation appropriate?

Not applicable

Are all the source data underlying the results available to ensure full reproducibility?

Partly

Is the study design appropriate and is the work technically sound?

Yes

Are the conclusions drawn adequately supported by the results?

Yes

Are sufficient details of methods and analysis provided to allow replication by others?

Partly

Reviewer Expertise:

Clinical Epidemiology, Systematic review, Research Waste, Setting priorities for research

I confirm that I have read this submission and believe that I have an appropriate level of expertise to confirm that it is of an acceptable scientific standard.

# Response

**Published:** 2021-07-02

**Authors:** Anne AnneCody

**Affiliations:** HRB, Ireland

Thank you for these insights, I am working to address these in V2.

# Response

**Published:** 2021-07-21

**Authors:** Anne AnneCody

**Affiliations:** HRB, Ireland

**In order to be able to reproduce the data, the readers need to know more about, "...initially each author collated relevant HRB materials, policies, or practices of which they were aware in their area of expertise." - Either how they identified/searched them or a list of these documents (if not all of them is possible, at least some examples of them).**

 Thank you for this useful suggestion. We have added the following text: 

 [The scope of the material to be included under each recommendation was agreed jointly] and included for example:

- HRB’s track record in supporting specific areas such as evidence synthesis, clinical trials or public and patient involvement;
- Recent extension of HRB function to include the secretariats for the Health Research Consent Declaration Committee linked to Irish data protection legislation and preparation for a National Research Ethics Committee for regulated medicinal products and medical devices;
- National policy activities such as co-leading of the National Research Integrity Forum and the National Open Research Forum, provision of an open publishing platform HRB Open Research, enabling the training of data stewards in host institutions via the GO FAIR Office, and requirement for Host Institutions to hold Athena Swan awards,
- Implementation activities regarding international initiatives such as the San Francisco Declaration on Research Assessment (DORA), the All Trials Campaign or the Ensuring Value in Research Funders’ Forum;
- Grant policies such as on Clinical Trials and Interventions Governance, Data Protection, Open Access, Data Management and Sharing, or Research Misconduct;
- Practices such as those identified in HRB Call Guidance Notes for each call including the requirement to present systematically gathered evidence, and reference to good practices such as use of core outcome sets, registration of protocols etc, selection of only international reviewers to avoid conflicts of interest, unconscious bias briefings and measures, external observers invited to selection panel meetings.

**In Table 1: It would be useful that in the self- assessment that you would differentiate between transparency around having criteria and process in place on how decisions are made (which a lot of funders do) versus transparency around how those process are implemented e.g. minutes of meeting or external observants in the room (which less often done by funders).**

 Reference added.

**Table 1, row 3 item (a): For this, "Requirement of systematically gathered evidence ((1) systematic identification of previous work, 2) critical appraisal, 3) synthesis of the evidence and 4) interpretation of findings) in growing number of schemes", is it possible to give a more accurate number than growing number of schemes or at least an estimate of how many schemes or applications that applies to. It will be also useful to highlight whether it is for researcher-driven grants or commission based one too or both?**

 We have added text: "in currently three investigator-led schemes with a total of approx. 120 applications." The HRB has few commissioned grants.

**Table 1, row 3 item (b): "Applications for clinical trials requests search of relevant registries" - Is that part of a systematic review or separate?**

 We have added text: "also". This is the the same context as the systematic review and ensures that the these registries are searched to avoid any ambiguity.

**Table 1, row 4 item (d): "HRB active in a number of relevant policy initiatives" - can you give example of one initiative?**

 We have added text: "[HRB active in a number of relevant policy initiatives] e.g. member of the EU Commission Expert Group on National Points of Reference on Scientific Information, funds a number of networks, implements the Science Europe Practical Guide to the Internal Alignment of Research Data Managements"

**Table 1, row 6: can you explain what "FAIR data steward training pilot" is? You talk about FAIR data in the text but don't explain what it is.**

 We have added text: "Enabled a mentoring and training scheme led by GOFAIR international Office to upskill existing staff in HRB host institutions as FAIR data stewards to apply the FAIR principles". Included link to FAIR principles

**Table 1, row 11: "...responsible for health-care systems, can help to improve the efficiency of clinical research be promoting integration of research in everyday clinical practice" - I assume it is improve the efficiency of clinical research "by" promoting integration?**

 Thank you for spotting this typo.

**Consistency in using the abbreviation - In this sentence, "HRB involved in Department of Health Group preparing Health Information Strategy Health Research Forum to be set up by DOH with national healthcare provider, HRB and other stakeholders", if you use DOH as abbreviation might be useful to add after department of health (DOH).**

 We have made this consistent, thanks for spotting.

**It wasn't clear to me how (HRB funding and dissemination activities) addressed item 11.**

 We have clarified with additional text: "HRB funding of healthcare interventions, health services research and population health research with associated knowledge transfer activities to inform practice. In schemes aimed at informing health policy, it is mandatory to include knowledge users as co-applicants."

# Peer review: Reviewer response for version 1

**DOI:** 10.21956/hrbopenres.14382.r29269

**Published:** 2021-04-26

**Authors:** Danielle Kemmer, Rory White

**Affiliations:** 1 Graham Boeckh Foundation, Montreal, QC, Canada

This paper describes a very important self-audit of Ireland's national health research funder. It emphasizes the critical role of funders in reducing waste in research, and provides detailed achievements to date and areas for improvement aligned with the recommendations made by the Lancet series in 2014.  

**Citations:**

 To complete the article, please include:

- A reference to the FAIR Guiding Principles ^1^.
- A reference or link for DORA.
- A link to the 10 Ensuring Value in Research Guiding Principles specifically tailored to funders.
- A reference or link to HRB Strategy detailing information about decision-making (Recommendation 2a).

**Methods:**

 In Recommendation 1, please provide information on how you define basic research (e.g. Health Research Classification System - Research Activities).

 What does ‘good overlap between authors’ mean? Please elaborate on differences in rating to provide a clear sense of the extent of agreement between authors.

**Other comments:**

 In Recommendation 4a, data on awarded HRB research grants should also be submitted to a grant data portal that consolidates grant data from various funding sources. This would greatly facilitate broader research funding analyses. We admit, however, that there is currently a scarcity of available public data repositories. 

 It would be helpful to provide a timeframe for the next iteration of HRB’s self-audit to monitor progress on areas of partial strength and areas for growth.

Is the work clearly and accurately presented and does it cite the current literature?

Partly

If applicable, is the statistical analysis and its interpretation appropriate?

Not applicable

Are all the source data underlying the results available to ensure full reproducibility?

Partly

Is the study design appropriate and is the work technically sound?

Yes

Are the conclusions drawn adequately supported by the results?

Yes

Are sufficient details of methods and analysis provided to allow replication by others?

Partly

Reviewer Expertise:

Research funding; research management; research priority setting; health research; grant stewardship; not-for-profit sector; philanthropy

We confirm that we have read this submission and believe that we have an appropriate level of expertise to confirm that it is of an acceptable scientific standard.

## References

[1] : The FAIR Guiding Principles for scientific data management and stewardship. *Sci Data* .2016;3: 10.1038/sdata.2016.18 160018 10.1038/sdata.2016.18 26978244PMC4792175

# Response

**Published:** 2021-07-02

**Authors:** Anne AnneCody

**Affiliations:** HRB, Ireland

Many thanks for your useful comments, Danielle, which I will strive to address in V2.

# Response

**Published:** 2021-07-21

**Authors:** Anne AnneCody

**Affiliations:** HRB, Ireland

**To complete the article, please include:**

- **Citations:**
- **A reference to the FAIR Guiding Principles ^1^ .**
- **A reference or link for DORA.**
- **A link to the 10 Ensuring Value in Research Guiding Principles specifically tailored to funders.**
- **A reference or link to HRB Strategy detailing information about decision-making (Recommendation 2a).**

Thank you, all added.

**Methods:**

**In Recommendation 1, please provide information on how you define basic research (e.g. Health Research Classification System - Research Activities).**

 Included link to HRB strategy with this definition.

**What does ‘good overlap between authors’ mean? Please elaborate on differences in rating to provide a clear sense of the extent of agreement between authors.**

 Thank you for this useful suggestion. We added the text: "For ten recommendations, all four authors individually came to the same rating. For the remaining seven recommendations, three authors individually came to the same rating, and one rated it with an adjacent rating (e.g. 2 instead of 1 or 1 instead of 2)".

**Other comments:**

**In Recommendation 4a, data on awarded HRB research grants should also be submitted to a grant data portal that consolidates grant data from various funding sources. This would greatly facilitate broader research funding analyses. We admit, however, that there is currently a scarcity of available public data repositories.**

 We fully agree, and will work with our researcher community on implementation when we become aware of suitable repositories.

**It would be helpful to provide a timeframe for the next iteration of HRB’s self-audit to monitor progress on areas of partial strength and areas for growth.**

 This is a good point, however we have not currently an agreed timeframe. Most likely the next self-audit will use the self-assessment tool the first author is developing with PCORI colleagues under the Ensuring Value in Research Funders' Forum rather than the framework used for this paper.
